# Personality Functioning and the Cortical Midline Structures – An Exploratory fMRI Study

**DOI:** 10.1371/journal.pone.0049956

**Published:** 2012-11-26

**Authors:** Stephan Doering, Björn Enzi, Cornelius Faber, Jens Hinrichs, Judith Bahmer, Georg Northoff

**Affiliations:** 1 Department of Psychoanalysis and Psychotherapy, Medical University of Vienna, Vienna, Austria; 2 Department of Psychiatry and Psychotherapy, University of Bochum, Bochum, Germany; 3 Department of Clinical Radiology, University of Münster, Münster, Germany; 4 Department of Psychosomatics and Psychotherapy, University of Münster, Münster, Germany; 5 Mind, Brain Imaging and Neuroethics, University of Ottawa Institute of Mental Health Research, Ottawa, Canada; University of Wuerzburg, Germany

## Abstract

**Objective:**

Recent neuroscience studies explored the neuronal mechanisms underlying our sense of self. Thereby the cortical midline structures and their anterior and posterior regions have been shown to be central. What remains unclear though is how both, self and cortical midline structures, are related to the identity of the self which is of central importance in especially personality disorders.

**Methods:**

Conducting an exploratory study with a dimensional approach, we here compared subjects with high and low level of personality functioning and identity integration as measured in a standardized way in fMRI during both, emotion- and reward-related tasks.

**Results:**

Low levels of personality functioning and identity integration were predicted by significantly decreased degrees of deactivation in the anterior and posterior cortical midline structures.

**Conclusions:**

Though exploratory our results show for the first time direct relationship between cortical midline structures and personality functioning in terms of identity integration. This does not only contribute to our understanding of the neuronal mechanism underlying self and identity but carries also major implications for the treatment of patients with personality disorders.

## Introduction

The self has recently become a focus of neuroscience. Several imaging studies compared self- and non-self-specific stimuli and observed major signal changes in the cortical midline structures (CMS) [1], [2], [3]. These studies seem to concern the degree of self-specificity associated with specific stimuli. While they leave open the neural correlates of our sense of self or what is often described as ‘mental self’ [4], [5]. One central component of the mental self is the temporal dimension. Our self extends across time from the past over the present to the future. Taken in such temporal sense, the self becomes associated with what earlier philosophers like John Locke described as ‘personal identity’. This raises the question for the neuronal mechanisms underlying identity which though remains to be explored.

In addition to the recruitment of the CMS during self-specific stimuli, recent studies also observed strong overlap between self-specific activity and resting state activity. Self-specific stimuli did not induce strong deviation from the resting state [3], [6], [7], [8]. This led to the suggestion that the resting state may be particularly implicated in constituting our sense of self. Since the resting state extends across time it may also provide a neural substrate for the transtemporal nature of our self, its identity. If so, one would expect the degree of deviation of task-related activity from the resting state in the CMS to be related to the identity. That though remains to be tested.

The concepts of self and identity can be identified in different ways either in a philosophical-psychological or psychodynamic sense. Philosophically and psychologically, the concept of identity describes the sameness of a particular person across time hence implying a diachronic aspect. While the concept of self concerns more the subjective experience of a person as a particular person, a sense of self indicating that a particular event, object, or person is part of that person at that instance in time. In contrast to the diachronic nature of the concept of identity, the concept of self has to be therefore understood more in a synchronic sense [1], .

Besides such philosophical-psychological definition, one may also determine both terms self and identity in a psychodynamic sense [9]. In his object relations theory, Kernberg assumes that very early in the development positive and negative experiences in relationships are internalized in a separate way of each other. These so-called (positive and negative) split-off internalized dyads are integrated to a holistic image of the self and significant others until the end of the third year of life [10]. These images are characterized by positive and negative aspects, shades of grey instead of black-and-white. Under conditions of adverse early experiences in relationships, this integration does not take place properly with an internal world of split-off internalized aspects of the self and others persisting instead. This state has been denominated as identity diffusion by Kernberg [10].

The clinical consequences of this core pathology can be subsumed under the heading of an impaired personality organization (i.e., personality functioning). In addition to an impaired ability to experience the self and others, the respective individuals are characterized by a low quality of object relations, primitive defense mechanisms, destructive aggressive impulses directed against the self and others, and impaired moral values.

Recently the concept of personality functioning moved into the focus of psychiatric diagnosis. The upcoming DSM-5 [11] provides the assessment of personality functioning as obligatory for the diagnosis of a personality disorder. The DSM-5 Levels of Personality Functioning Scale will cover the domains self (identity, self-direction) and interpersonal (empathy, intimacy). The DSM-5 defines identity as an ongoing awareness of a unique self with the maintenance of role-appropriate boundaries, consistent and self-regulated positive self-esteem, with accurate self-appraisal, and the capability of experiencing, tolerating, and regulating a full range of emotions [11]. A similar severity measure will be included into ICD-11 [12]. Patients with personality disorders, especially borderline patients, suffer from severe problems in these functions, this state was described as identity diffusion by Kernberg [10]. A longstanding tradition of the assessment of personality functioning exists in psychoanalysis and the psychodynamic therapies [10], [13], [14]. During the last decade a number of standardized instruments were developed for the assessment of this dimension (for review see [15]). One of these, the Structured Interview of Personality Organisation (STIPO [16]) was employed in this study for the dimensional assessment of identity.

The aim of our study was to exploratorily test the relationship between personality functioning in terms of identity integration and cortical midline structures. For that purpose, we investigated a cohort of healthy subjects and patients with borderline personality disorder. Both groups were assessed with regard to psychiatric disorders as well as personality functioning and underwent fMRI during two tasks, emotions and reward.

## Methods

### Study Design

The study was approved by the ethics committees of the Universities of Münster and Magdeburg, Germany. After a detailed explanation of the study, all subjects gave their written informed consent.

All subjects were interviewed by means of Structured Clinical Interview for DSM-IV (SCID-I and -II [17], [18]) and the Structured Interview of Personality Organisation (STIPO [16]). In addition, well-established neuropsychological tests were completed: the LPS-3 (German: Leistungsprüfsystem-3 [19]) and MWT-A (German: Mehrfach-Wortschatz-Intelligenztest A [20]) as measurements of general intelligence and the Beck Depression Inventory (BDI [21]). Handedness was determined by the Edinburgh Inventory [22]. After the assessments, patients underwent functional MRI.

### Subjects

17 female subjects without psychiatric (except two cases of insect phobia), neurological or medical illnesses (age 26.41±6.97 years, range 19–49, 2 left handed, 15 right-handed) and 17 thoroughly matched female patients suffering from borderline personality disorder (age 28.88±9.34 years, range 20–48 years, 2 left handed, 15 right-handed) were investigated.

Patients yielded 4.53±1.84 DSM-IV axis I diagnoses and 3.47±1.51 DSM-IV axis II diagnoses assessed by means of SCID-I and -II. One patient (5.9%) had one comorbid axis I diagnosis, two (11.8%) had two, three (17.6%) had three, five (29.4%) had five, four (23.5%) had six, and two (11.8%) had seven axis I diagnoses. Including BPS, one patient (5.9%) had one axis II diagnosis, three (17.6%) had two, seven (41.2%) had three, two (11.8%) had four, one (5.9%) had five, and three (17.6%) had six axis II diagnoses.

All healthy subjects were free from psychiatric medication. Eleven patients were on regularly psychiatric medication (only antidepressants: 5 patients; only neuroleptics: 1 patient; antidepressants and neuroleptics: 4 patients; antidepressants, neuroleptics, and sedatives: 1 patient).

### Structured Interview of Personality Organization (STIPO)

The STIPO [16] was developed by Otto F. Kernberg and colleagues at the Cornell University New York. It represents the structured version of the Structural Interview that was developed by Kernberg in the 1980s [10], [23]. The instrument is based on the above mentioned object relations theory of Kernberg [10]. The interview contains 100 items that are addressed by one or more specific questions. The single-item rating is made by the interviewer on a three point scale with operationalized descriptions for each level: 0 = pathology absent, 1 = minor impairment, 2 = significant to severe impairment. The interview covers seven domains: (1) identity, (2) object relations, (3) primitive defences, (4) coping/rigidity, (5) aggression, (6) moral values, and (7) reality testing and perceptual distortions. The identity dimension contains three sub-dimensions: (A) capacity to invest, (B) sense of self (coherence and continuity, self-valuation), and (C) sense of others.

Guided by operationalized anchors each domain and subdomain is rated on a five-point scale with “1” standing for the absence of pathology and “5” indicating most severe impairment of personality functioning.

An overall rating is generated from the ratings of the dimensions. Six different levels of personality organization (i.e., personality functioning) are provided for the overall rating: (1) normal, (2) neurotic 1, (3) neurotic 2, (4) borderline 1, (5) borderline 2, and (6) borderline 3.

Satisfactory reliability and validity of the English as well as of the German version of the instrument have been demonstrated [24], [25]. Moreover, it has served as an outcome variable in a treatment study with borderline patients and demonstrated significant change of personality organization after one year in patients treated with Transference-Focused Psychotherapy (TFP) [26] compared to a control condition [27]. The English version has recently been shortened to 87 items. Both, English and German versions, are freely available in the internet.

### Experimental Paradigm

Before scanning, all subjects completed a short practice version of the task to familiarize with the experiment.

The fMRI scanning session was divided into three scanning runs. In the first scanning run, subjects had to perform a modified version of the well-established monetary incentive delay task (for details see [28], [29]), requiring that the subjects press a button with the index finger of their right hand within a certain time of a target image (a black square in the centre of the screen) being displayed. The length of this time period was determined in accordance with the average reaction time obtained in the pre-scan trial run, allowing the difficulty of the task to be modulated according to the individual's ability, and varied between 0.2 s and 0.35 s. Furthermore, we wanted to ensure that in approximately 60% of all trials the required response was successful. Prior to this target image being displayed, a symbol indicating what the possible outcomes of the task would be – either reward, punishment, or no-outcome – was shown for 2 s, followed by a 2.25–2.75 s anticipation period. The trial type indicator took the form of a black circle with a small white circle within it at one of the cardinal points. Each position represented a different trial type, e.g. reward, punishment, or no outcome ( = control) trials.

In reward trials, completing the task successfully resulted in the subject winning €1, whilst failure meant that they would neither win nor lose anything. During punishment trials, a response within the required time period resulted in the subject neither winning nor losing money, whilst an unsuccessful response resulted in €1 being deducted from their total. Finally, in no-outcome trials no money was either won or lost, regardless of whether the subject responded within the required time period or not. Subjects were, however, instructed to still respond to the cue as quickly as possible. In total, 40 reward and punishment trials and 30 no outcome trials were displayed in a pseudo-random order. Each trial was followed by a feedback stage during which the subject was informed of the outcome. The amount of money won or lost in the preceding trial was displayed, along with the running total for their winnings, for a period of 1.65 s. Trials were separated by a 2.5–3.5 s inter-trial interval, during which the same light coloured cross as that shown during the anticipation period was displayed.

In the second and third run a modified version of the above described monetary incentive delay task was presented. Briefly, simultaneously with the presentation of the outcome indicating symbol, i.e. reward, punishment or no outcome, an emotional picture was presented, resulting in a 3-by-3 factorial design. The pictures were categorized according to their affective valence (positive, negative, or neutral emotion). The total number of conditions was balanced to ensure the calculation of valid contrasts (e.g., 30 reward trials with positive emotion, 30 reward trials with negative emotion, and 20 reward trials with neutral emotion). All different time periods (cue, anticipation, target, feedback, and inter-trial interval) were equal to the above mentioned monetary incentive delay task without emotional modulation.

In all functional runs, a separate baseline condition (duration 4.5 to 5.5 s), was presented pseudo-randomized after approximately ten trials. All participants received the amount of money they had earned during the whole experiment. (A detailed overview concerning the applied experimental procedure is given in [29].)

### fMRI Data Acquisition

Functional data was collected using a 3-Tesla whole body MRI system (Philips Achieva) equipped with a Philips transmit and receive head coil. Using a midsagittal scout image, a stack of 32 T2*-weighted single-shot echo-planar images (sshEPI) was aligned parallel to the bicommissural plane. During each functional run 540 whole brain volumes were acquired (matrix 64×64, field-of-view 230×230 mm^2^, spatial resolution: 3.59×3.59×3.60 mm^3^, TE = 30 ms, TR = 2000 ms, flip angle 90°). Prior to the functional scanning session, a high-resolution, T1-weighted anatomical 3D gradient echo scan was acquired for each subject (matrix 256×153×80, FOV 256×204×160 mm^3^, spatial resolution 1×1.33×2 mm reconstructed to 0.5×0.5×1 mm^3^, TE = 3,4 ms, TR = 6.9 ms, flip angle 9°, 2 averages).

### fMRI Data Analysis

The functional data were pre-processed and statistically analysed using SPM5 (Wellcome Department of Cognitive Neuroscience, University College London, UK; http://www.fil.ion.ucl.ac.uk) and MATLAB 6.5.1 (Mathworks Inc, Natick, MA, USA). The first five volumes were discarded due to saturation effects. After slice timing and correction for between-scan motion artefacts by realignment to the first volume, the anatomical scan was co-registered to a mean functional image. The normalization was generated by warping the subject's anatomical T1-weighted scan on the T1-template provided by SPM5 (MNI stereotactic space) and applying these parameters to all functional images. The images were resampled to a final voxel size of 3×3×3 mm^3^ and smoothed with an isotropic 8 mm full-width half-maximum Gaussian kernel. The time-series fMRI data were filtered using a high pass filter and cut-off of 100 s.

For the monetary incentive delay task (1st run), all relevant conditions, i.e. anticipation of reward, anticipation of punishment, anticipation of no outcome, their feedback phase according to task performance (success vs. no success) and the baseline condition were modelled, resulting in ten conditions. Concerning the interaction task (2nd and 3rd run), all possible combinations of the conditions ‘task’ (reward, punishment and no outcome) and ‘emotion’ (positive, negative and neutral), and the baseline condition were modelled as regressors. Additionally, the six realignment parameters were entered as regressors of no interest. A statistical model for each subject was computed by convolving a canonical hemodynamic response function with the above mentioned design matrix [30] and followed the General Linear Model approach [31]. Regionally specific condition effects were tested by employing linear contrasts for each subject and different conditions. Since we were mainly interested in the so-called cortical midline structures, we specified the contrast [baseline>anticipation of no outcome] in each subject. The resulting contrast images were entered in a second level random effects analysis by calculating an one-sample t-test encompassing all subjects. Unless otherwise indicated a voxel-wise false discovery rate correction [32] was performed to control for multiple testing. Only activations surviving p[FDR] <0.05 for an extent of k >30 voxel were reported.

Using sphere shaped regions of interest (ROI; radius 5 mm) centered upon the peak voxel within each area of interest, beta-values for each condition were extracted and transformed into percent signal change using the Marseille Region of Interest Toolbox (MarsBaR; http://marsbar.sourceforge.net/) software package [33]. The above mentioned ROI analysis was focused on the major components of the cortical midline structures, i.e., the sub- and pregenual anterior cingulate cortex (SACC, PACC), the ventro- und dorsomedial prefrontal cortex (VMPFC, DMPFC), the supragenual and midorsal anterior cingulate cortex (MACC), the posterior cingulate cortex (PCC), and the retrosoplenial cortex (RSC). All further statistical analyses (t-tests for dependent and independent samples, Spearman's rank correlation, Shapiro-Wilk test) were calculated using the software package SPSS 13 (SPSS Inc., Chicago, USA).

## Results

### STIPO Results

The STIPO overall ratings were distributed as follows: normal: 10 (29.4%), neurotic 1: 4 subjects (11.8%), neurotic 2: 3 subjects (8.8%), Borderline 1: 5 subjects (14.7%), Borderline 2: 11 subjects (32.4%), and Borderline 3: 1 patient (2.9%).

For group comparison the study population was divided in two subgroups according to the level of personality organization, i.e. personality functioning, as measured by the STIPO overall rating. 17 subjects showed a high personality organization (HPO; normal, neurotic 1 and 2) in contrast to 17 subjects showed a low personality organization (LPO; Borderline 1–3). All healthy subjects showed a high personality organization, whereas all Borderline patients belong to the low personality organization group.

All STIPO sub-dimensions differed significantly between the two groups. The identity rating was 1.41±0.51 in the high organized group, and 3.59±0.51 in the low organized group, (group comparison: t = 12.51, df = 32, p<.001) (Table 1). Such grouping, thus, represents a dimensional approach in orientation on the level of personality organization across the distinction between healthy subjects and Borderline patients. Such dimensional approach must be distinguished from the usually pursued categorical approach that presupposes the categories of healthy subjects and Borderline patients.

**Table 1:** Characteristics of the study population.

	high personality organization (n = 17)	low personality organization (n = 17)	statistics^1^
age [years] (mean ± SD)	26.41±6.97	28.88±9.34	p = 0.389
age [range in years]	19–49	20–48	-
handedness [right/left]	15/2	15/2	-
intelligence [LPS-3] (mean ± SD)	120.18±9.44	113.06±12.16	p = 0.066
intelligence [MWT-A] (mean ± SD)	120.76±12.69	112.12±12.78	p = 0.056
depression [BDI] (mean ± SD)	1.71±2.93	32.71±9.17	p<0.001
**STIPO dimensions** (mean± SD)			
identity	1.41±0.51	3.59±0.51	p<0.001
object relations	1.24±0.56	3.06±0.75	p<0.001
primitive defence	1.35±0.61	3.82±0.73	p<0.001
coping and rigidity	1.53±0.72	3.88±0.86	p<0.001
aggression	1.12±0.33	3.35±0.70	p<0.001
moral values	1.12±0.33	2.41±0.87	p<0.001
reality testing	1.0^2^	2.35±0.70	p<0.001
personality organization overall rating	1.53±0.72	4.76±0.56	p<0.001

1 t-test for independent samples with 32 degrees of freedom;

2 all subjects showed a score of 1 concerning the dimension reality testing.

Abbreviations: SD: standard deviation; LPS-3: Leistungsprüfsystem-3; MWT-A: Mehrfach-Wortschatz-Intelligenztest A; BDI: Beck Depression Inventory; STIPO: Structured Interview of Personality Organization.

### fMRI Results

The contrast [baseline>anticipation of no outcome] revealed a consistent set of cortical midline structures commonly associated with the resting state or default mode network, i.e. the left subgenual anterior cingulate cortex, the bilateral dorsomedial prefrontal cortex, the bilateral dorsal and medial cingulate cortex and the bilateral retrosplenial cortex. In addition, we were able to detect significant activations located in limbic and paralimbic structures like for instance the bilateral amygdala and the bilateral parahippocampal gyrus (Table 2).

**Table 2:** Significant signal changes for the contrast [baseline>anticipation of no outcome].

region	coordinates [MNI]	p-value^1^	t-value	z-value
left subgenual ACC	−3, 27, −6	0.001	4.64	4.04
left DMPFC	−6, 57, 39	<0.001	5.36	4.51
right DMPFC	15, 48, 42	0.003	3.96	3.55
left dorsal cingulate cortex	−9, −39, 42	<0.001	7.16	5.52
right dorsal cingulate cortex	6, −36, 42	<0.001	5.33	4.49
right medial cingulate cortex	3, −9, 48	0.003	4.16	3.70
left retrosplenial cortex	−6, −54, 18	<0.001	7.97	5.91
right retrosplenial cortex	9, −51, 18	<0.001	9.00	6.35
left amygdala	−18, −3, −18	<0.001	5.87	4.82
right amygdala	21, −3, −18	0.001	4.61	4.02
left parahippocampal gyrus	−24, −21, −18	0.003	3.87	3.48
right parahippocampal gyrus	24, −15, −18	0.001	4.81	4.16

1 Initial threshold: p[FDR]<0.05, k>30.

Abbreviations: ACC: anterior cingulate cortex; DMPFC: dorsomedial prefrontal cortex.

**Figure 1:**
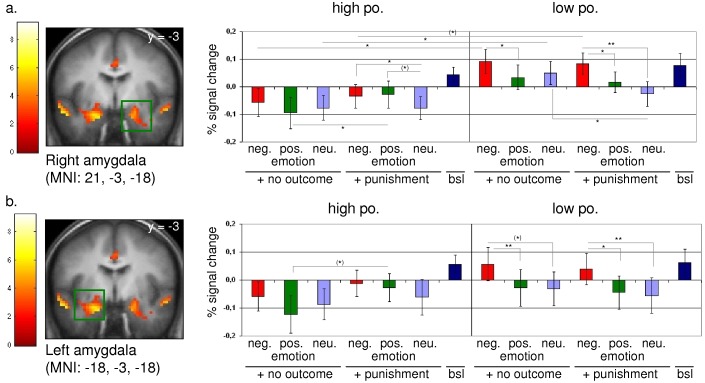
Statistical parametric map and percent signal change derived from the contrast [baseline>anticipation of no outcome], *p*[FDR]<0.05, *k*>30. (a.) Neuronal response for the right amygdala (MNI: 21, −3, −18). (b.) Neuronal response for the left amygdala (MNI: −18, −3, −18). ** p<0.01; * 0.05<p<0.01; ^(^*^)^ 0.1<p<0.05. Error bar: Standard Error of the Mean (SEM).

A more fine-grained analysis revealed deficits in emotion processing in subjects with low personality organization (LPO) located in the right amygdala. In comparison to subjects showing high personality organization (HPO), LPO subjects showed an enhanced neuronal response regarding the condition ‘no outcome+negative emotion’ (LPO vs. HPO: t_indep.;32_ = −2.210; p = 0.034) and ‘no outcome+neutral emotion’ (LPO vs. HPO: t_indep.;32_ = −2.067; p = 0.047). Moreover, LPO subjects showed a general hyperreactivity to negative emotional stimuli in the very same region (‘no outc.+neg. emotion’ vs. ‘no outc.+pos. emotion’: t_paired;16_ = 2.701; p = 0.016), notably under simultaneous presentation of a punishing cue (‘punish.+neg. emotion’ vs. ‘punish.+neutr. emotion’: t_paired;16_ = 2.3; p = 0.035; ‘punish.+neg. emotion’ vs. ‘punish.+pos. emotion’: t_paired;16_ = 3.921; p = 0.001). The above outlined finding of a hyperreactivity to negative emotional stimulation is confirmed by our findings regarding the left amygdala (‘no outc.+neg. emotion’ vs. ‘no outc.+pos. emotion’: t_paired;16_ = 2.939; p = 0.009; ‘punish.+neg. emotion’ vs. ‘punish.+neutr. emotion’: t_paired;16_ = 3.079; p = 0.007; ‘punish.+neg. emotion’ vs. ‘punish.+pos. emotion’: t_paired;16_ = 2.167; p = 0.046) (Figure 1).

**Figure 2:**
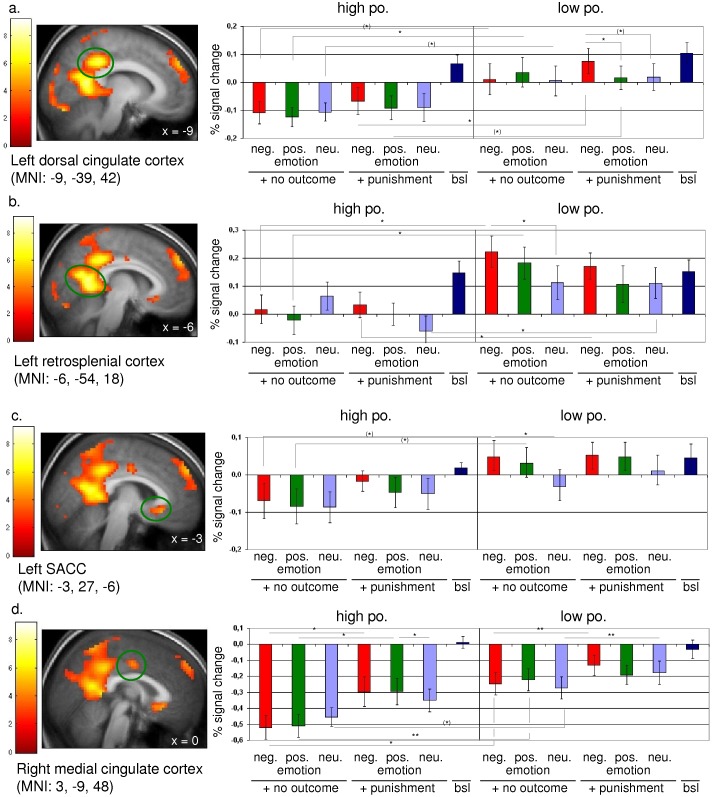
Statistical parametric maps and percent signal change derived from the contrast [baseline>anticipation of no outcome] in subjects showing high or low personality organization. (a.) Left dorsal cingulate cortex (MNI: −9, −39, 42), (b.) Left retrosplenial cortex (MNI: −6, −54, 18), (c.) Left subgenual anterior cingulate cortex (SACC) (MNI: −3, 27, −6), and (d.) Right medial cingulate cortex (MNI: 3, −9, 48). *p*[FDR]<0.05, *k*>30. ** p<0.01; * 0.05<p<0.01; ^(^*^)^ 0.1<p<0.05. Error bar: Standard Error of the Mean (SEM).

Concerning the ROI in the cortical midline structures, the HPO subjects showed strong deactivations, i.e. negative BOLD response (NBR), in relation to the baseline condition in several regions, whereas LPO subjects showed a disturbed neuronal differentiation with positive BOLD response (PBR) in the left DCC (LPO vs. HPO: ‘no outc.+pos. emotion’: t_indep.;32_ = −2.542; p = 0.016; ‘punish.+neg. emotion’: t_indep.;32_ = −2.177; p = 0.037) and the left retrosplenium (LPO vs. HPO: ‘no outc.+neg. emotion’: t_indep.;32_ = −2.705; p = 0.011; ‘no outc.+pos. emotion’: t_indep.;32_ = −2.688; p = 0.011; ‘punish.+neg. emotion’: t_indep.;32_ = −2.107; p = 0.043; ‘punish.+neu. emotion’: t_indep.;32_ = −2.217; p = 0.034). In the right medial cingulate cortex, LPO subjects showed a reduced NBR regarding the no outcome condition under simultaneous emotional stimulation (LPO vs. HPO: ‘no outc.+neg. emotion’: t_indep.;32_ = −2.682; p = 0.011; ‘no outc.+pos. emotion’: t_indep.;32_ = −2.924; p = 0.006). In addition, the notion of hyperreactivity for negative emotional stimulation in LPO subjects was confirmed for the punishment condition in the left DCC (‘punish.+neg. emotion’ vs. ‘punish.+pos. emotion’: t_paired;16_ = 2.331; p = 0.033), and for the no outcome condition regarding the left retrosplenium (‘no outc.+neg. emotion’ vs. ‘no outc.+neutr. emotion’: t_paired;16_ = 2.992; p = 0.009) and the left SACC (‘no outc.+neg. emotion’ vs. ‘no outc.+neutr. emotion’: t_paired;16_ = 2.422; p = 0.028) (Figure 2).

### Correlations of STIPO Identity and fMRI

**Figure 3:**
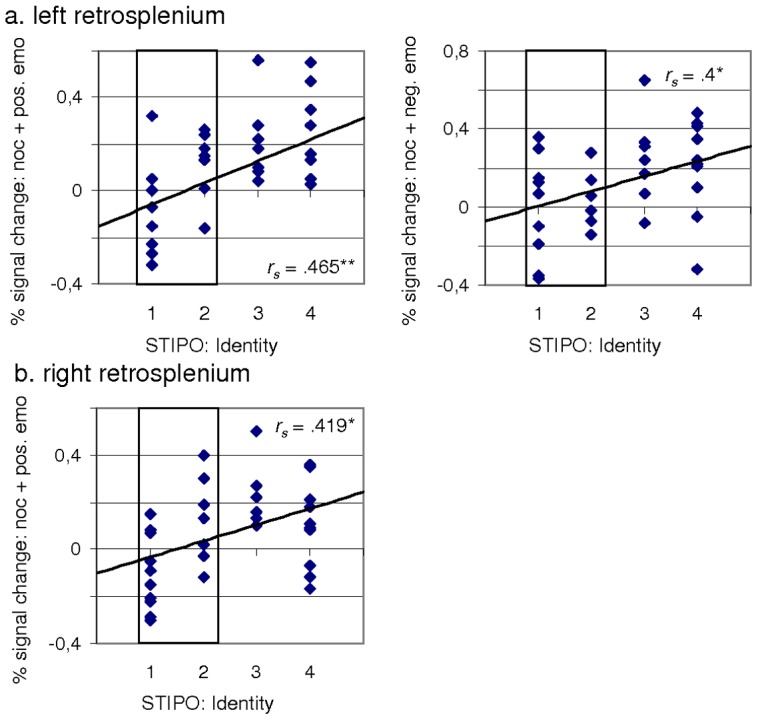
Correlation between the STIPO dimension ‘identity’ and the fMRI signal change. (a.) Left retrosplenium (MNI: −6, −54, 18), and (b.) Right retrosplenium (MNI: 9, −51, 18). Subjects showing a high personality organization according to the STIPO overall rating are highlighted in blue. Spearman correlation. *r_s_*: Spearman rho. * 0.05<p<0.01; ** p<0.01.

Since cortical midline structures play a crucial role in self-related processing, Spearman correlations between the fMRI signal change derived from the above mentioned ROI and the STIPO dimension ‘identity’ were calculated. Spearman's rank correlation was used since the Shapiro-Wilk test showed significant results regarding the identity dimension of the STIPO. We detected significant positive correlations between the identity domain and the conditions ‘no outcome+negative emotion’ (r_s_ = .400, p = 0.019) and ‘no outcome+positive emotion’ (r_s_ = .465, p = 0.006) regarding the left retrosplenial cortex (MNI coordinates: −6, −54, 18). In the right retrosplenial cortex (MNI coordinates: 9, −51, 18) a significant positive correlation between ‘identity’ and ‘no outcome+positive emotion’ (r_s_ = .419, p = 0.014) was observed (see Figure 3). When the HPO and the LPO groups were analysed separately, the correlations remained significant in the HPO only: Left retrosplenial cortex and ‘no outcome+positive emotion’ (r_s_ = .562, p = 0.019), right retrosplenial cortex and ‘no outcome+positive emotion’ (r_s_ = .561, p = 0.019).

## Discussion

We here investigated for the first time the relationship between personality functioning and cortical midline structures relying on a dimensional approach. Our exploratory results show a significant relationship between signal changes in various cortical midline structures - more precisely in the left and right retrosplenial cortex - and the level of personality functioning in the domain of identity. Better personality functioning in terms of identity integration was associated with a stronger deactivation in the CMS during the emotional component of the applied interaction paradigm. Though exploratory, this is the first study that investigates neuronal predictors of personality functioning and more specifically identity as core component of our self. This has not only major neuronal implications, but also contributes clinically to psychiatry and psychotherapy.

These results have a number of implications. First of all, a better personality functioning in the realm of identity seems to be facilitated by deactivation of CMS during task-related activity in emotion processing. The CMS (default mode network, DMN) are known to show high levels of neural activity during both, resting state and self-related processing [3], [6], [7], [8]. The degree of deactivation of this set of regions during task-related activity can then be taken as indirect sign of the neuronal reactivity of the DMN to particular stimuli and tasks (see also [5]). Lower degrees of deactivation as observed in our subjects with low personality organization may, thus, consecutively be assumed to indicate decreased neuronal reactivity of the DMN to changes in its activity level during task-related demands. While such assumption of decreased neuronal reactivity in the DMN remains tentative at this point, future studies may want to investigate other more direct measures of resting state activity like functional connectivity and low frequency fluctuations. Given our current results, one would expect abnormalities in both, functional connectivity and low frequency fluctuation power in especially the CMS in subjects with lowly organized identity levels.

Why do the apparent abnormalities in the CMS as core nucleus of the DMN impact the identity? The identity of a person is a central part of his or her self. More specifically, identity introduces a temporal component that spans and extends the self from the past over the present to the future. Recent studies demonstrate especially the CMS to be strongly implicated in both retrospection and prospection [34], [35], [36], [37]. Moreover, studies by d'Argembeau *et al.* [38], [39], [40] demonstrated the neural activity in the CMS to be related to the retrospection and prospection of the self. Our finding of identity being related to the degree of deactivation in core regions of the DMN during task-related activity extends these observations by applying for the first time a direct and well established measure for identity: Since identity presupposes the trans-temporal constitution of self across past, present, and future, our results complement the studies showing CMS involvement in temporal extension, i.e., prospection and retrospection. They thus bridge the gap from the CMS and the self to the identity of the latter. Future studies may directly target and investigate the interaction between prospection/retrospection and identity of the self.

To put it differently, we determined identity as diachronic concept that operates across time. While the self, e.g., the experience or sense of self, is more confined to one particular point in time, thus, being synchronic rather than diachronic. Previous results showed involvement of especially the anterior midline structures in the self and its synchronic component [3]. In contrast, studies showing identity and its diachronic component to be related to the midline regions have not been demonstrated yet. Hence, our study is the first one showing that the diachronic aspects of our self, e.g., identity, may also be mediated by the midline regions especially the posterior midline regions. Future studies may thus want to directly compare synchronic and diachronic aspects, e.g., self and identity, to test whether they can be differentiated with regard to anterior and posterior midline regions. Moreover, the direct interaction and inter-dependence between self and identity and between synchronic and diachronic components may be of strong interest in especially psychiatric patients where both may differ. Hence, our study does not only show for the first time the association of identity with the midline structures but raises also some important questions that in general may be highly relevant to better understand the brain and its midline structures in psychological regard.

This close association of identity and its diachronic component with the posterior midline structures is well in accordance with the central role of the precuneus and the retrosplenuim in autobiographical memory retrieval. These regions have been associated with retrospection and thus past, as also documented in their central role in the retrieval of episodic and autobiographical memory [41]. While anterior regions like the anterior cingulate cortex are rather associated with prospection and thus the future [34], [35] as also evidenced by the involvement in anticipation (see the present results and also for instance [42]). Since low and high levels of identity showed clearly different degrees of deactivation in these regions, one may assume that a lowly functioning identity may be related to difficulties of putting the own self into the broader temporal context, i.e., future and past. This is further supported by the fact that our paradigm required subjects to anticipate thus implying a strong temporal dimension as outlook into the future. One may consecutively assume that the recruitment of the CMS may allow to put our self into the temporal flow from the past over the presence to the future. Such ‘temporalization’ of the self may for instance be disturbed in psychiatric disorders like depression [9], [43], [44] and may henceforth serve as a potential target for future more specific psychotherapeutic approaches [9], [45], [46].

For clinical investigations in psychiatry and psychotherapy this study provides a paradigm that allows for the assessment of aspects of personality functioning. This might be employed, e.g., in predictor or outcome studies of psychotherapy.

From a conceptual point of view, our results support Kernberg's emphasis on identity impairment as core pathology in borderline personality disorder and other conditions with an impairment of personality functioning. As a consequence, future psychotherapeutic treatments might specifically need to address personality functioning and identity for a sustainable improvement not only of symptoms, but also of psychosocial adaptation in this group of patients.

Given the low number of participants this is clearly an exploratory study. Furthermore one may want to criticize that our patients were mostly on medication and showed severe psychopathology. In our previous study on the same patients [29] we demonstrated that medication was of minor impact on the results generated by means of the monetary incentive delay task. Finally, one may also be estranged by the dimensional approach pursued which goes against the usually presupposed categorical approach with its clear-cut distinction between healthy and non-healthy groups. However, personality functioning in general and identity in particular are regarded as continuous variables rather than discrete discontinuous variables (see e.g. [11]).

We here demonstrate for the first time the direct relationship between identity and deactivation in the cortical midline structures as core nucleus of the default-mode network. Increased levels of deactivation during task-related activity as for instance emotions and reward predict a higher level of personality functioning in general and identity in particular. This has not only major implications for our understanding of the neural basis of self and identity but also for the development of novel psychotherapeutic strategies in the future.
